# Preoperative Uric Acid-to-Albumin Ratio as a Predictor of Postoperative Atrial Fibrillation After Cardiac Surgery

**DOI:** 10.21470/1678-9741-2024-0377

**Published:** 2025-09-26

**Authors:** Atilla Koyuncu, Cennet Yıldız, Ersan Oflar, Hasan Ali Sinoplu, Atakan Arpaç, Bilgin Bayraktar, Esra Dönmez, Sevgi Özcan, Mustafa Ozan Gürsoy, Fatma Nihan Turhan Çağlar, Ali Aycan Kavala

**Affiliations:** 1 Department of Cardiology, Bakırköy Dr Sadi Konuk Education and Research Hospital, Istanbul, Turkiye; 2 Department of Cardiology, Bağcılar Education and Research Hospital, Istanbul, Turkiye; 3 Department of Cardiology, Izmir Ataturk Education and Research Hospital, Izmir, Turkiye; 4 Department of Cardiovascular Surgery, Bakırköy Dr. Sadi Konuk Education and Research Hospital, Istanbul, Turkiye

**Keywords:** Albumin, Cardiac Surgery, Atrial Fibrillation, Uric Acid, Inflammation.

## Abstract

**Introduction:**

Postoperative atrial fibrillation (POAF), the pathophysiology that includes inflammation and oxidative stress, is associated with increased hospital length of stay, mortality, and complications. The uric acid-to-albumin ratio reflects the inflammatory status of the body. We sought to evaluate whether there is an association between POAF and uric acid-to-albumin ratio in patients undergoing cardiac surgery.

**Methods:**

Five hundred forty-three patients who developed POAF and 166 patients who did not formed our control and study groups, respectively. Patients who had an episode of atrial fibrillation lasting > 30 seconds were considered to have POAF. The uric acid-to-albumin ratio was calculated for each patient.

**Results:**

Patients who developed POAF were older; had higher rates of hypertension, carotid artery disease, left atrial diameter, urea, creatinine, uric acid, and C-reactive protein levels; and had lower hemoglobin and albumin levels. The uric acid-to-albumin ratio of patients with and without POAF was 1.65 ± 0.63 and 1.26 ± 0.39, respectively (P < 0.001). Compared with uric acid and albumin, uric acid-to-albumin ratio had the highest area under the curve for predicting POAF (0.681, 0.449, and 0.702, respectively). Age and hemoglobin concentration were predictors of POAF. Although uric acid and albumin did not reach statistical significance for predicting POAF, the uric acid-to-albumin ratio had predictive value for the development of POAF.

**Conclusion:**

The ability of the uric acid-to-albumin ratio to predict POAF in cardiac surgery patients and its nonnegligible benefits justify its use in clinical practice.

## INTRODUCTION

**Figure:**
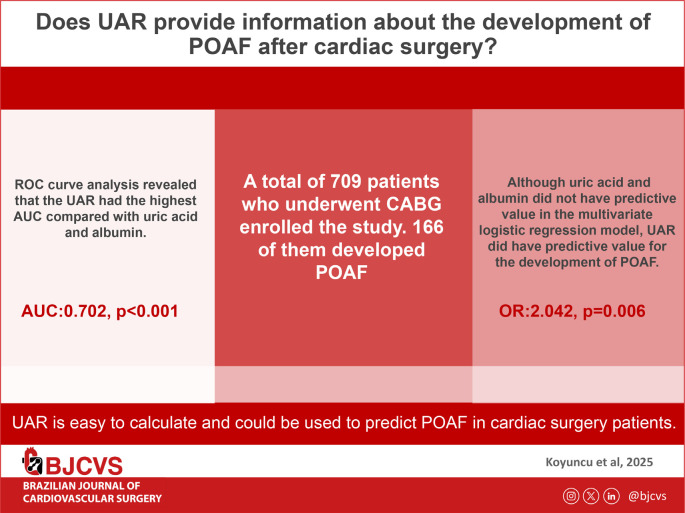

**Table:** 

Abbreviations, Acronyms & Symbols				
ACEI	= Angiotensin converting enzyme inhibitor		Hgb	= Hemoglobin
AF	= Atrial fibrillation		LA	= Left atrial
ARB	= Angiotensin receptor blocker		LDL-C	= Low-density lipoprotein cholesterol
AUC	= Area under the curve		LVEF	= Left ventricular ejection fraction
BMI	= Body mass index		OR	= Odds ratio
CABG	= Coronary artery bypass grafting		POAF	= Postoperative atrial fibrillation
CI	= Confidence interval		ROC	= Receiver operating characteristic
COPD	= Chronic obstructive pulmonary disease		TC	= Total cholesterol
CRP	= C-reactive protein		TSH	= Thyroid stimulating hormone
HDL-C	= High-density lipoprotein cholesterol		UAR	= Uric acid-to-albumin ratio
HgA1c	= Hemoglobin A1c			

Postoperative atrial fibrillation (POAF), or new-onset atrial fibrillation after surgery, is fairly common after both cardiac and noncardiac surgeries, with rates of 20 to 40% and 10 to 20%, respectively^[1]^. Although it usually manifests as a brief and self-limited attack, its presence lengthens the hospital stay and increases the risk of recurrent atrial fibrillation (AF) and mortality^[2]^. POAF is thought to be stimulated by transient factors that act on the preexisting atrial substrate. Histological examinations of atrial tissue from patients who developed POAF revealed left atrial fibrosis, cardiomyocyte hypertrophy, and cellular degeneration^[3,4]^. Atrial injury predisposes patients to the pathogenesis of POAF by creating inhomogeneous and anisotropic conduction in the atrial tissue^[1]^. Similarly, the activation of epicardial fibroblasts alters connexin 40 and 43 levels, resulting in slowing of conduction^[5]^. Inflammation and oxidative stress have been suggested as key mechanisms for the occurrence of POAF^[6]^. By activating both cellular and noncellular elements of the blood, cardiopulmonary bypass itself is a major source of the inflammatory response^[7]^. Surgery-induced ischemia resulting in a decrease in mitochondrial respiration leads to increased generation of reactive oxygen species and a decrease in antioxidant activity^[8]^. In addition, ischemia‒reperfusion injury causes the production of proinflammatory molecules and endothelial cell and leukocyte activation. It has been reported that the onset of POAF coincides with the peak of white blood cell count during hospitalization^[9]^. Elevated levels of interleukin-2 and -6 and C-reactive (CRP) protein have been found in patients with POAF^[10]^. The inhibition of POAF by the administration of corticosteroids is further evidence of the role of inflammation in the pathogenesis of POAF^[11]^. Finally, autonomic system alterations, electrolyte imbalances, advanced age, hypertension, left atrial dilatation, chronic obstructive pulmonary disease, diabetes mellitus, and chronic renal failure are other factors associated with increased risk of POAF^[12]^.

The prevention of POAF, which is still a major problem today, has become an important goal, and it is clear that many efforts have been made in this regard. To this end, several indicators have been proposed, such as traditional risk scores, which are widely used. As the role of inflammation in the pathogenesis of POAF has been suggested, a substantial number of inflammatory biomarkers have been shown to have predictive value for POAF risk^[13]^. In addition, several formulas/indices have been proposed that include these biomarkers, and it has been suggested that the combination of more than one biomarker may better reflect the inflammatory status^[14]^. The neutrophil-to-lymphocyte ratio, the CRP-to-albumin ratio, and the systemic immune-inflammation index, which includes neutrophil, platelet, and lymphocyte counts, are some of these indices that have predictive value for the occurrence of POAF^[15-17]^. The uric acid-to-albumin ratio (UAR), which has gained importance in recent years, combines both the inflammatory activity of uric acid and the anti-inflammatory activity of albumin. It provides prognostic information for various clinical conditions, such as transcatheter aortic valve implantation, the prediction of atrial fibrillation after ST-elevation myocardial infarction, coronary artery disease severity, and acute kidney injury^[18-21]^. We hypothesized that our study would shed light on whether UAR provides information on the development of atrial fibrillation after cardiac surgery and would highlight a pathophysiological mechanism as well as do a risk stratification of this patient group.

## METHODS

The records of patients who underwent coronary artery bypass grafting between January 1, 2020, and January 1, 2022, at two tertiary hospital centers were retrospectively reviewed. A total of 1,076 files were obtained during this period. The exclusion criteria for study participation were as follows: (1) presence of atrial fibrillation; (2) unavailable or missing medical records; (3) severe hepatic or renal dysfunction; (4) thyroid hormone abnormalities; (5) uric acid lowering treatment; (6) infectious diseases; (7) malignancy; (8) amiodarone therapy; (9) valvular heart disease; and (10) emergency surgery. After application of the exclusion criteria, 709 patients remained, of whom 166 developed POAF and 543 did not develop POAF. Ethical approval was given from the local ethics committee (approval number: 2023-09-02), and written informed consent was obtained from each patient before study entry. The study was conducted in accordance with the principles of the Helsinki Declaration.

Our study and control groups were composed of patients with POAF (n = 166) and without POAF (n = 543). Information regarding the demographic and clinical variables was obtained from the hospital recording system. Echocardiographic examination of the patients was performed before the operation in accordance with the current guidelines^[22]^. For all patients, the left ventricular ejection fraction (LVEF) was calculated via the modified Simpson method. All patients underwent on-pump surgery with median sternotomy in the setting of moderate systemic hypothermia. The mean intensive care unit stay was 3.46 ± 2.78 days.

Patients were considered to have POAF if they had an atrial fibrillation episode lasting > 30 seconds or required medical or electrical cardioversion treatment after surgery. Blood samples that were collected 24 hours before the operation were used for the analyses. Complete blood cell counts were determined via an autoanalyzer. Serum uric acid and albumin concentrations were determined via a Roche Diagnostics Cobas 8000 c502 analyzer (Roche Holding AG, Basel, Switzerland). The UAR was calculated by dividing uric acid by serum albumin.

### Statistical Analysis

The normality of the data was assessed via Q-Q plots. Continuous and categorical data are expressed as the means/standard deviations and numbers/percentages, respectively. Comparisons between groups that developed POAF and those that did not were made via Student's *t*-test or the Mann-Whitney U test. Categorical variable comparisons were made via the chi-square test. A receiver operating characteristic (ROC) curve was used to estimate the cutoff value of the UAR for the development of POAF. Univariate logistic regression was used to identify predictors of POAF. The clinical variables that were significant for prediction were included in the multivariate logistic regression analysis. Statistical significance was accepted as a *P*-value < 0.05.

## RESULTS

**Fig. 1:**
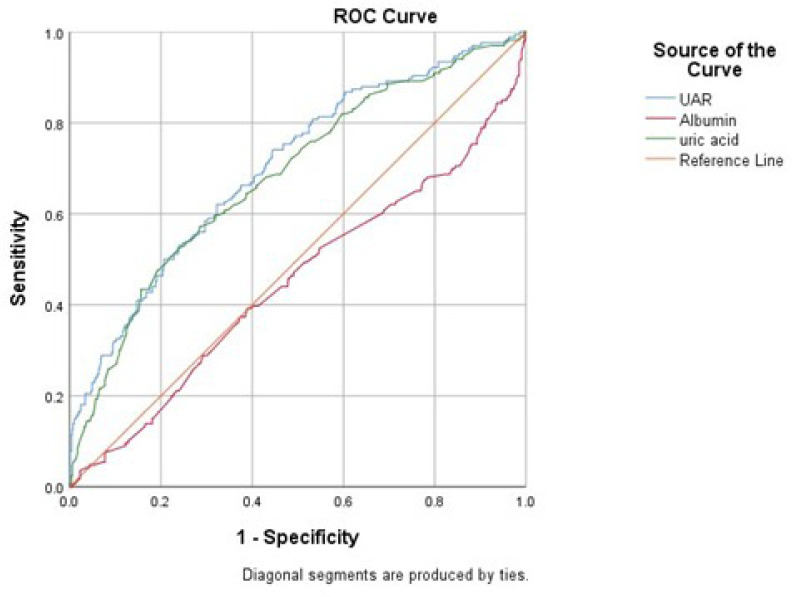
Receiver operating characteristic (ROC) curve analysis of uric acid-to-albumin ratio (UAR) for predicting postoperative atrial fibrillation.

The mean age of the patients was 60.24 ± 10.19 years, the mean LVEF was 51.01 ± 8.77, 158 (22.3%) patients were female, 359 (56%) were hypertensive, and 294 (41.5%) were diabetic. Patient characteristics are shown in Table 1. A comparison of patients who developed POAF with those who did not revealed that patients who developed POAF were older (59.14 ± 10.20 years *vs.* 63.80 ± 9.31 years), had higher rates of hypertension (53.6% *vs.* 63.9%), carotid artery disease (22.2% *vs.* 31.8%), greater left atrial diameter (35 ± 4.00 mm *vs.* 36.05 ± 4.21 mm), urea (36.04 ± 18.63 mg/dL *vs.* 43.88 ± 23.05 mg/dL), creatinine (0.94 ± 0.67 mg/dL *vs.* 1.19 ± 0.88 mg/dL), uric acid (5.14 ± 1.47 mg/dL *vs.* 6.27 ± 1.75 mg/dL), and CRP levels (16.73 ± 26.75 mg/dL *vs.* 24.71 ± 37.68 mg/dL), and lower hemoglobin (12.66 ± 1.76 g/dL *vs.* 12.04 ± 1.76 g/dL) and albumin (4.20 ± 1.83 g/dL *vs.* 3.99 ± 0.57 g/dL) levels. The UAR of patients with POAF was found to be 1.65 ± 0.63, and that of patients without POAF was found to be 1.26 ± 0.39, indicating a statistically significant difference (*P* < 0.001). We found no differences between the two groups with respect to sex, body mass index, presence of diabetes mellitus, chronic obstructive pulmonary artery disease, hyperlipidemia, medication use, LVEF, glucose, hemoglobin A1c, thyroid-stimulating hormone levels, lipid profile, leukocyte, lymphocyte, monocyte, neutrophil, platelet counts, and extracorporeal circulation time. A comparison of patients with and without POAF is shown in Table 2. ROC curve analysis was performed to determine the cutoff values for uric acid, albumin, and UAR, which revealed that UAR had the highest area under the curve (AUC) for predicting POAF (0.681, 0.449, and 0.702, respectively) (Figure 1). Compared with the uric acid and albumin levels, the UAR also had the highest specificity (77.2%, 73.2%, and 51.5%, respectively). The results of the ROC curve analysis with the cutoff, sensitivity, and specificity values of uric acid, albumin, and UAR are shown in Table 3.

**Table 1:** Clinical characteristics of the study population (n = 709).

Age (years)	60.24 ± 10.19
Sex (n, %)	
Female	158 (22.3)
Male	551 (77.7)
BMI (kg/m^2^)	28.36 ± 4.57
Hypertension (n, %)	397 (56)
Diabetes mellitus (n, %)	294 (41.5)
COPD (n, %)	107 (15.1)
Carotid artery disease (n, %)	173 (24.4)
Hyperlipidemia (n, %)	412 (58.1)
Statin (n, %)	448 (63.3)
ACEI-ARB (n, %)	358 (50.5)
Beta blocker (n, %)	370 (52.2)
LVEF (%)	51.01 ± 8.77
LA diameter (mm)	35.32 ± 4.08
Glucose (mg/dL)	144.20 ± 64.70
HgA1c (%)	6.75 ± 1.85
Hgb (g/dL)	12.52 ± 1.78
Urea (mg/dL)	38.38 ± 20.34
Creatinine (mg/dL)	1.00 ± 0.73
Uric acid (mg/dL)	5.45 ± 1.69
Albumin (g/dL)	4.15 ± 1.62
CRP (mg/dL)	18.25 ± 29.29
LDL-C (mg/dL)	113.26 ± 45.34
HDL-C (mg/dL)	39.56 ± 9.93
Triglyceride (mg/dL)	164.15 ± 105.74
TC (mg/dL)	184.46 ± 52.97
Leukocyte count (10^3^/µL)	9.34 ± 5.36
Lymphocyte count (10^3^/µL)	2.10 ± 0.83
Monocyte count (10^3^/µL)	0.64 ± 0.23
Neutrophil count (10^3^/µL)	6.09 ± 2.78
Platelet count (10^3^/µL)	243.03 ± 74.48
TSH (mIU/L)	1.82 ± 1.81
UAR	1.35 ± 0.49

ACEI=angiotensin converting enzyme inhibitor; ARB=angiotensin receptor blocker; BMI=body mass index; COPD=chronic obstructive pulmonary disease; CRP=C-reactive protein; HDL-C=high-density lipoprotein cholesterol; HgA1c=hemoglobin A1c; Hgb=hemoglobin; LA=left atrial; LDL-C=low-density lipoprotein cholesterol; LVEF=left ventricular ejection fraction; TC=total cholesterol; TSH=thyroid stimulating hormone; UAR=uric acid-to-albumin ratio

**Table 2:** Comparison of two groups.

	Control group (n = 543)	Study group (n = 166)	*P*-value
Age (years)	59.14 ± 10.20	63.80 ± 9.31	< 0.001
Sex (n, %)			0.135
Female	114 (21)	44 (26.5)	
Male	429 (79)	122 (73.5)	
BMI (kg/m^2^)	28.82 ± 4.51	28.61 ± 4.86	0.547
Hypertension (n, %)	291 (53.6)	106 (63.9)	0.020
Diabetes mellitus (n, %)	215 (39.6)	79 (47.6)	0.067
COPD (n, %)	75 (13.9)	31 (18.8)	0.135
Carotid artery disease (n, %)	120 (22.2)	52 (31.8)	0.015
Hyperlipidemia (n, %)	(58.4)	(57.1)	0.779
Statin (n, %)	(65.3)	(58.4)	0.229
ACEI-ARB (n, %)	(49.2)	(53.2)	0.463
Beta blocker (n, %)	(53.4)	(49.6)	0.498
LVEF (%)	51.73 ± 8.77	51.43 ± 8.79	0.897
LA diameter (mm)	35 ± 4.00	36.05 ± 4.21	0.034
Glucose (mg/dL)	140.74 ± 64.99	152.51 ± 63.54	0.073
HgA1c (%)	6.74 ± 1.90	6.81 ± 1.68	0.200
Hgb (g/dL)	12.66 ± 1.76	12.04 ± 1.76	0.001
Urea (mg/dL)	36.04 ± 18.63	43.88 ± 23.05	0.002
Creatinine (mg/dL)	0.94 ± 0.67	1.19 ± 0.88	0.004
Uric acid (mg/dL)	5.14 ± 1.47	6.27 ± 1.75	< 0.001
Albumin (g/dL)	4.20 ± 1.83	(3.99 ± 0.57	0.043
CRP (mg/dL)	16.73 ± 26.75	24.71 ± 37.68	0.024
LDL-C (mg/dL)	112.31 ± 46.24	115.52 ± 43.24	0.325
HDL-C (mg/dL)	39.71 ± 10.23	39.18 ± 9.19	0.527
Triglyceride (mg/dL)	161.37 ± 98.19	170.80 ± 122.18	0.143
TC (mg/dL)	183.13 ± 54.52	187.65 ± 49.18	0.094
Leukocyte count (10^3^/µL)	9.19 ± 2.89	9.17 ± 3.92	0.168
Lymphocyte count (10^3^/µL)	2.15 ± 0.85	2.00 ± 0.76	0.497
Monocyte count (10^3^/µL)	0.65 ± 0.23	0.63 ± 0.24	0.167
Neutrophil count (10^3^/µL)	6.09 ± 2.66	6.10 ± 3.08	0.416
Platelet count (10^3^/µL)	239.42 ± 67.22	251.63 ± 89.22	0.725
TSH (mIU/L)	1.73 ± 1.63	2.25 ± 2.52	0.223
Extracorporeal circulation time (min)	146.35 ± 38.86	151.92 ± 64.22	0.679
UAR	1.26 ± 0.39	1.65 ± 0.63	< 0.001

ACEI=angiotensin converting enzyme inhibitor; ARB=angiotensin receptor blocker; BMI=body mass index; COPD=chronic obstructive pulmonary disease; CRP=C-reactive protein; HDL-C=high-density lipoprotein cholesterol; HgA1c=hemoglobin A1c; Hgb=hemoglobin; LA=left atrial; LDL-C=low-density lipoprotein cholesterol; LVEF=left ventricular ejection fraction; TC=total cholesterol; TSH=thyroid stimulating hormone; UAR=uric acid-to-albumin ratio

**Table 3:** Receiver operating characteristic curve analysis of uric acid, albumin, and UAR for prediction of postoperative atrial fibrillation.

	AUC	*P*-value	95% CI	Cutoff	Sensitivity	Specificity
Uric acid	0.681	< 0.001	0.634 - 0.729	5.98	54.2	73.2
Albumin	0.449	0.027	0.396 - 0.502	4.12	46.4	51.5
UAR	0.702	< 0.001	0.655 - 0.748	1.56	51.2	77.2

AUC=area under the curve; CI=confidence interval; UAR=uric acid-to-albumin ratio

Univariate logistic regression revealed that age (odds ratio [OR]: 1.048), carotid artery disease (OR: 1.631), hypertension (OR: 1.530), left atrial diameter (OR: 1.066), hemoglobin concentration (OR: 0.822), creatinine (OR: 1.483), uric acid (OR: 1.513), albumin (OR: 0.568), CRP (OR: 1.008), and UAR (OR: 5.311) were independent predictors of the occurrence of POAF (Table 4). We performed two multivariate logistic regression models to identify the predictors of POAF. Model A and Model B included uric acid, albumin, and UAR, respectively, in addition to the other variables found in the univariate analysis (Table 5). Our analysis revealed that in both models, age and hemoglobin concentration were predictors of POAF. Although uric acid and albumin did not reach statistical significance in Model A, UAR had predictive value for the development of POAF. Main findings of the study are illustrated in the Central Figure.

**Table 4:** Univariate logistic analysis for prediction of postoperative atrial fibrillation.

	*P*-value	OR	95% CI
Age	< 0.001	1.048	1.030 - 1.068
Carotid artery disease	0.016	1.631	1.096 - 2.427
Hypertension	0.030	1.530	1.069 - 2.190
LA diameter	0.027	1.066	1.007 - 1.128
Hemoglobin	< 0.001	0.822	0.744 - 0.908
Creatinine	0.001	1.483	1.166 - 1.896
Uric acid	< 0.001	1.513	1.346 - 1.700
Albumin	0.002	0.568	0.397 - 0.813
CRP	0.004	1.008	1.002 - 1.013
UAR	< 0.001	5.311	3.537 - 7.975

CI=confidence interval; CRP=C-reactive protein; LA=Left atrial; OR=odds ratio; UAR=uric acid-to-albumin ratio

**Table 5:** Multivariate logistic analysis for prediction of postoperative atrial fibrillation.

MODEL A			
	*P*-value	OR	95% CI
Age	0.006	1.039	1.011 - 1.068
Carotid artery disease	0.766	1.083	0.639 - 1.836
Hypertension	0.494	0.830	0.485 - 1.418
LA diameter	0.158	1.049	0.982 - 1.121
Hemoglobin	0.040	0.856	0.737 - 0.993
Creatinine	0.634	0.896	0.570 - 1.408
Uric acid	0.237	1.107	0.936 - 1.309
Albumin	0.537	0.903	0.654 - 1.248
CRP	0.246	1.004	0.997 - 1.011
CI=confidence interval; CRP=C-reactive protein; LA=left atrial; OR=odds ratio			

MODEL B			
	*P*-value	OR	95% CI
Age	0.008	1.038	1.010 - 1.067
Carotid artery disease	0.799	1.071	0.631 - 1.820
Hypertension	0.483	0.826	0.484 - 1.410
LA diameter	0.233	1.042	0.974 - 1.114
Hemoglobin	0.042	0.859	0.742 - 0.995
Creatinine	0.473	0.836	0.513 - 1.363
CRP	0.378	1.003	0.996 - 1.010
UAR	0.006	2.042	1.227 - 3.397

CI=confidence interval; CRP=C-reactive protein; LA=Left atrial; OR=odds ratio; UAR=uric acid-to-albumin ratio

## DISCUSSION

Our results showed that in addition to risk factors such as older age and lower hemoglobin concentrations, UAR is another risk factor for POAF, adding to the evidence that inflammation is involved in the development of POAF.

The role of inflammation in the pathogenesis of POAF has been extensively studied. Preoperative factors as well as the surgical inflammatory response influence this pathophysiology^[1,2]^. Chronic low-grade inflammation has been shown to play a role in both the development and maintenance of atrial fibrillation^[6]^. The contact of blood with cardiopulmonary bypass circuit surfaces activates leukocytes and induces the release of various cytokines and reactive oxygen species^[7]^. In addition, promising results have been obtained with the use of several anti-inflammatory and antioxidant agents, such as colchicine, acetylcysteine, and statins, for the prevention of POAF, supporting the role of inflammation in POAF development^[13]^. The UAR combines both uric acid and albumin concentrations and provides information about the global inflammatory and oxidative status of the body. Selçuk et al. reported that the UAR has value in predicting the occurrence of new-onset atrial fibrillation, with an OR of 6.95 in patients with ST-elevation myocardial infarction. In their study, UAR had a greater AUC value than did CRP, uric acid, and albumin alone^[19]^. Karataş et al. followed 170 patients with atrial fibrillation who underwent cryoballoon catheter ablation for a median of 22 months. They reported that patients with UAR ≥ 1.67 were associated with a 2.70-fold increased risk of atrial fibrillation^[23]^. Although various inflammatory markers have been studied and found to have predictive value in patients with POAF^[13-17]^, to our knowledge, this is the first study to evaluate UAR in patients undergoing cardiac surgery. In our study, UAR had moderate diagnostic accuracy, with an AUC of 0.702, and moderate to high specificity (77.2%) for predicting POAF. In addition, unlike uric acid and albumin, these parameters had a predictive value for the development of POAF according to multivariate analysis. This better value of UAR probably results from the incorporation of two biomarkers, uric acid and albumin, one inflammatory biomarker and the other anti-inflammatory biomarker, into a single biomarker. The use of both methods may provide a more accurate and integrated measure of inflammatory activity, which is one of the major factors in the pathophysiology of POAF^[24]^. Various clinical risk models have been proposed that are based on epidemiologic data^[25,26]^. In light of our findings and previous data, incorporating variables reflecting inflammatory markers into these risk models might improve their accuracy.

In our opinion, other findings from the study deserve some discussion. There is a wide range of incidences of POAF in the literature, from 20% to as high as 40%^[27,28]^. The reason for such a wide range may lie in the definition of POAF; some studies described it as an episode of atrial fibrillation lasting > 30 seconds, whereas others described it as lasting > 5 - 60 minutes^[29,30]^. Some studies included patients who experienced atrial fibrillation in the first three days after surgery, whereas others included patients for the entire hospital stay. In the present study, episodes of atrial fibrillation lasting > 30 seconds during the entire hospital stay were considered POAF. We found that the incidence of POAF was 23.4%, which is consistent with previous data.

Several risk factors have been implicated in the development of POAF, including older age, heart failure, male sex, and smoking^[26]^. In our study, age and lower hemoglobin concentrations were predictors of POAF. Although other clinical factors, such as carotid artery disease, left atrial diameter, hypertension, and higher creatinine and CRP concentrations, were statistically significant predictors of POAF according to the univariate model, their significance was lost in the multivariate models. With advancing age, atrial remodeling occurs with an increase in fibrotic atrial tissue, which is associated with heterogeneous atrial conduction and a predisposition to atrial fibrillation^[31]^. It has been shown that there is a nonlinear relationship between POAF and age, with a maximum percentage of 80 years after 55 years of age^[32]^. By generating ischemia of both atrial and conduction tissues, increasing adrenergic activation and altering the electrophysiological properties of the heart, anemia may induce atrial fibrillation in this patient group. We found that lower hemoglobin concentrations were linked to increased POAF risk^[33,34]^.

### Limitations

The limitations of our study were as follows: (1) its design was retrospective; (2) long-term follow-up of the patients was not performed, so we could not draw conclusions about the prognostic effect of UAR; and (3) all the covariates that might have an effect on the development of POAF could not be evaluated.

## CONCLUSION

Our results suggest that UAR, an inflammatory biomarker, has predictive value for the occurrence of POAF in patients undergoing cardiac surgery. It is very easy to calculate and could be used in this group of patients.

## Data Availability

The authors declare that the data will be available upon request to the authors.
